# Backflow Effect Enabling Fast Response and Low Driving Voltage of Electrophoretic E-ink Dispersion by Liquid Crystal Additives

**DOI:** 10.1038/s41598-019-50382-y

**Published:** 2019-09-27

**Authors:** Ya-Di Zhang, Wen-Jie Hu, Zhi-Guang Qiu, Jia-Zhe Xu, Ming-Yang Yang, Yi-Fan Gu, Jin-Xin Cao, Peng Chen, Gui-Shi Liu, Bo-Ru Yang

**Affiliations:** 1 State Key Laboratory of Optoelectronic Materials and Technologies, Guangdong Province Key Laboratory of Display Material and Technology, and School of Electronics and Information Technology, Sun Yat-Sen University, Guangzhou, 510006 China; 2 Guangdong Provincial Key Laboratory of Optical Fiber Sensing and Communications, College of Science & Engineering, Jinan University, Guangzhou, 510632 China

**Keywords:** Liquid crystals, Liquid crystals

## Abstract

Electrophoretic display encountered several challenges towards high frame rate applications, such as long response time and high driving voltage. In this study, liquid crystal additive doping can simultaneously increase the response speed by 2.8 times and reduce the driving voltage to half of the initial value of electrophoretic dispersion. The backflow effect of liquid crystal, which induces an inversely electrorheological effect and facilitates the reverse micelles’ dielectrophoretic separation, was suggested to be the main reason for the performance improvement. The proposed method is facile and effective which shows promising potential for fast response and low power consumption e-paper applications.

## Introduction

Electrophoretic display (EPD) offers several advantages over conventional liquid crystal display (LCD) and organic light-emitting display (OLED) in terms of lower power consumption, higher sunlight visibility, and better reading experiences with less eyestrain. EPD has been therefore widely used in E-reader, advertising board, electrical shelf label, luggage labels, and smart medicine containers^1–4^. The superior features of EPD results from its display media, the electrophoretic dispersion, also known as electronic ink (E-ink). E-ink is made of electrophoretic particles with positive charges and negative charges in non-polar solution. The particles with opposite charge can be separated to achieve different gray level depending on the applied electric field. Because of the inherent scattering effects of E-ink particles, the image has no glare effect, and thus render better ambient contrast ratio^5^.

Although the EPD, as a reflective display, can provide good visual perception as printed papers, and conveniently download the image content with much lighter weight^6^, the long response time and high driving voltage precluded its application in high frame rate display. Given the attractive properties of EPD, there have been many studies done on the reduction of the response time and driving voltage. Kao *et al*.^7^ studied the variation of the response latency under different image retention time and demonstrated that some states in activation phase of particles could be removed to improve the response time of EPD without any degradation of performance. Bai *et al*.^8^ proposed an improved driving waveform, wherein the image was reset to white or black according to the gray scale of the previous image away from light or dark state, achieving the shortest route for the updating of gray image. Wang *et al*.^9^ used four kinds of image update mode corresponding to different types of image content to improve the response time. However, additional driving or updating waveform modification is of limited effectiveness. Another effective method is to employ a suspending liquid with low viscosity^10^, which can essentially increase electrophoretic mobility but deteriorate bistability at the meantime. A more feasible strategy is to realize viscosity reduction of the suspending liquid only while applying voltage and viscosity recovering after removing voltage, known as inversed electrorheological (IER) effect^11^. It has been reported that the spontaneous rotation of small particles under applied voltage in a liquid, also known as Quincke rotation, could render a viscosity reduction by driving the surrounding liquid and exhibiting an IER effect^12–14^. We noted that, according to Ericksen-Leslie continuum theory^15^, LC molecules can rotate under applied electric field and induce hydrodynamic motion (backflow effect). It is envisaged that the backflow effect of LC which has been widely used in micro actuators^16–18^ and micro-particle manipulation^19,20^, could induce an IER effect.

In this paper, we sought to increase the mobility of electrophoretic particles by utilizing the LC backflow effect. A cell device of electrophoretic display was fabricated to demonstrate the IER behavior. The effects of LC materials on electro-optical property of electrophoretic dispersion were investigated by characterizing the response time, driving voltage, spectral intensities, and current. The experimental results demonstrated that LC doping could greatly reduce both the response time and driving voltage of EPD.

## Methods

### Materials preparation and device fabrication

The pristine electrophoretic dispersion consisted of 22.5 wt% of white particles, 7.5 wt% of black particles, 0.31 wt% of charge control agent (CCA), 0.6 wt% of thicker and nonpolar solvent. A nematic LC E7 (PhiChem Corporation) with a positive dielectric anisotropy Δε = 13.8 was added into the electrophoretic dispersion. The mixture was subjected to ultrasonication for 1 hour and overnight shaking to achieve uniform suspensions.

**Figure 1:**
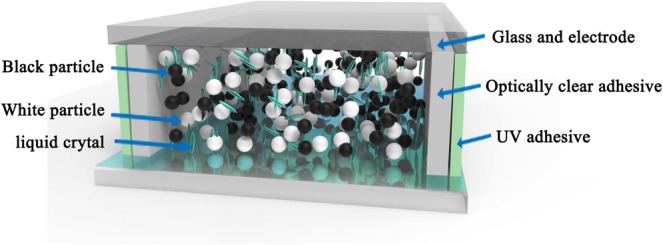
Structure of the electrophoretic dispersion test cell.

A test cell was prepared to contain the electrophoretic dispersion. As shown in Fig. 1, two strips of optically clear adhesive (OCA) with a thickness of 50 μm was attached to the two side ends of the indium tin oxide (ITO) substrate as a spacer to maintain the cell gap. After laminating the substrate with another ITO glass, two sides of the cell were sealed by an ultraviolet (UV) adhesive (NOA65, provided by Norland). The dispersion was filled into the test cell by capillary force, and the cell was then sealed completely to avoid solvent volatilization. Cell gap was measured by using a spectrophotometer (Tsushima Evolution 220) and calculated with the result of 50.5 ± 1 μm, which is consistent with the thickness of OCA.

### Characterization on electro-optical response of EPD

The electro-optical response was monitored by an optical setup where the incident light was illuminated from a LED, collimated by a convex lens, projected onto the EPD device, reflected or absorbed according to the EPD’s display states, and then finally detected by a photo detector. The reflectance was measured by the spectrophotometer. A standard waveform combination was used to drive the EPD device, consisting of three phases: shaking, resetting, and driving. Shaking phase was used to erase the previous image and activate the particles. Resetting phase was used to reset the image to black or white state as a reference to ensure the origin state is consistent each time before driving phase. The EPD device was driven with ±15 V and the response time was defined as the time between 10% and 90% of optical intensity. Furthermore, a (PWM) waveform was utilized to characterize the driving voltage and activated voltage of the EPD device. The PWM waveform has a fixed pulse width of 750 ms and gradually increasing amplitude from 0 V to 20 V with 0.5 V step. The negative voltage of −20 V was used to reset the image. The spectral intensities of the non-polar solvent doping with LC was measured using confocal Raman spectroscopy. The current of the EPD device was measured using a digital sourcemeter (Keithley, 2400).

## Results

### Response time and reflectance

**Figure 2:**
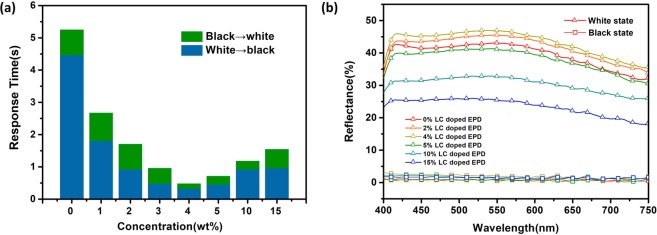
(**a**) The response time and (**b**) reflectance of electrophoretic dispersion doped with a different concentration of liquid crystal (E7).

The doping of E7 gave rise to a significant reduction in the response time of the EPD device. As shown in Fig. 2a, the response time decreased with the increase of E7 concentration until E7 has reached to 4 wt%. The optimal white to black (WTB) and black to white (BTW) response time were 320 ms and 163 ms under 15 V of driving voltage, respectively, which was about ten times faster than the one with pristine dispersion. In addition, the reflectance of white state of the EPD device increased slightly with increment of E7 concentration to 4 wt% (Fig. 2b). The result suggests that the doping of E7 might improve the particles electrophoretic velocity under the applied electric field. The particles with higher velocity could move more thoroughly and accumulated more tightly near the surface of electrodes which led to the enhancement of reflectance. However, when the concentration of E7 exceeded 4 wt%, the reflectance of the white state started to decrease. This phenomenon was caused by the particle aggregation induced by the thin double layer under applied voltage due to LC over doping which would be explained in detail later.

### Driving voltage and activated voltage

**Figure 3:**
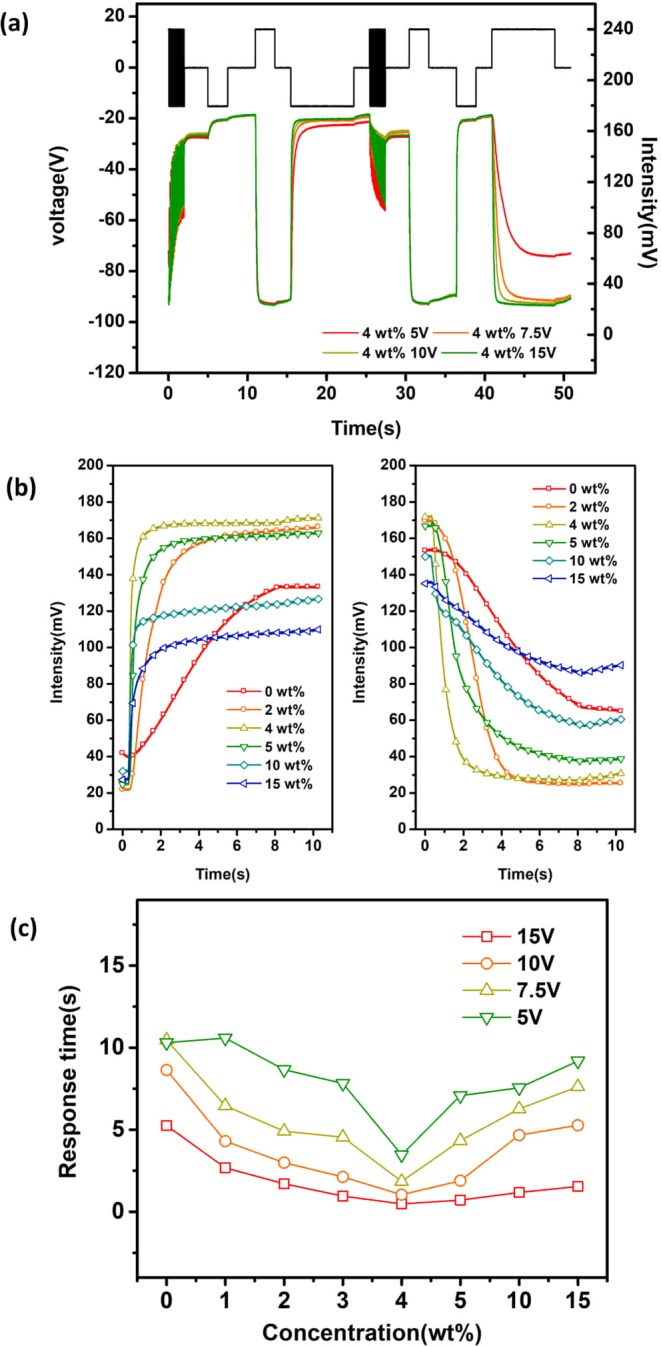
The electro-optical response of electrophoretic dispersion (**a**) doped with 4 wt% LC under different driving voltages and (**b**) doped with different concentrations of LC under 7.5 V driving voltage. (**c**) The response time of electrophoretic dispersion doped with different concentrations of LC under different voltages.

Being able to drive the electrophoretic particles with a low voltage is crucial for EPD. Figure 3a displays the performance of the LC-doping (4 wt%) device under different driving voltages from 3 V to 15 V. The performance of the pristine electrophoretic dispersion under different driving voltages from 3V to 15V is shown in the Supplementary Figure S1. It could be seen that the electrophoretic dispersion doped with 4 wt% LC achieved approximately the same dynamic range when the driving voltage decreased from 15 V to 7.5 V, while the device of pristine dispersion exhibited significant degradation of 53.5% in dynamic range (Fig. 3b). In addition, the response time of each concentration at different voltages was illustrated in Fig. 3c. The response time of electrophoretic dispersion with 4 wt% LC at 7.5 V is not only smaller than that of pristine dispersion at 7.5 V, but also 2.8 times smaller than that at 15 V. The result suggested that doping with LC could simultaneously achieve faster response and better white and black states with lower driving voltage.

**Figure 4:**
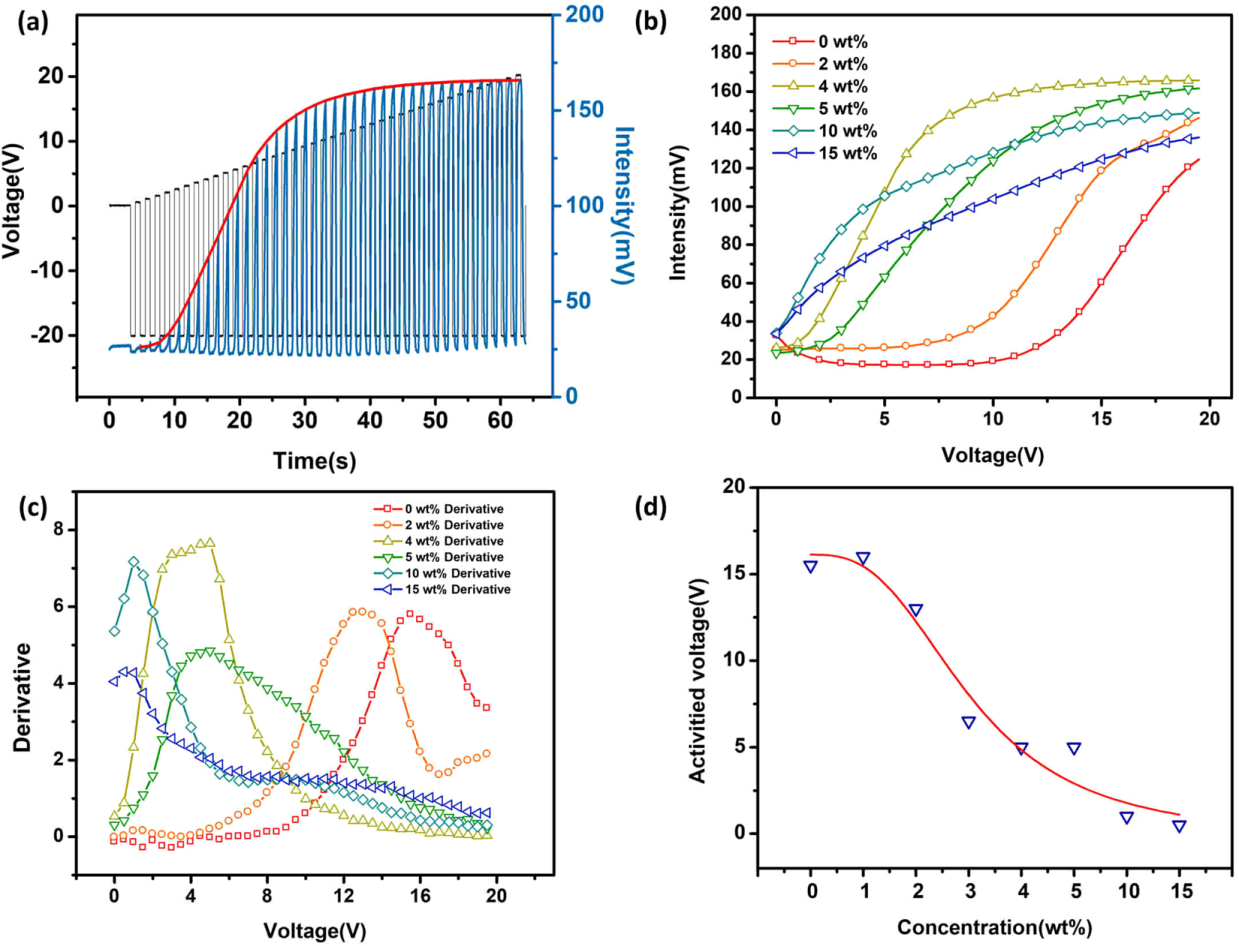
The driving measurement of electrophoretic dispersion at different LC doped concentration. (**a**) Waveform of driving voltage and optical response of dispersion doped with 4 wt% LC. (**b**) The curve of optical response peak at each voltage steps. (**c**) The derivative of the response peak curve and (**d**) activated voltage.

To further clarify the LC-doping effect on the driving voltage, the cells were driven by a PWM waveform of increasing amplitude at a 0.5 V step. As illustrated in Fig. 4a, the optical intensity of dispersion doped with 4 wt% LC increased exponentially after a few pulses and became saturated at 11.5 V. The peak of intensity for each driving step was extracted for different LC concentrations and plotted as Fig. 4b. Both the growth rate and the maximum value of intensity increased as the increment of LC concentration and saturated at about 4 wt% of LC concentrations. The intensities of 10 wt% and 15 wt% of LC doping dispersion were higher than that of 4 wt% at low driving voltage, but the maximum intensities of the two samples were still much lower than that of 4 wt%. Therefore, 4 wt% concentration of LC is optimal in aspects of response time and contrast ratio.

In order to probe the variation trend of the response velocity more accurately, the derivative of the intensity (Fig. 4b) with respect to the driving voltage was calculated, as illustrated in Fig. 4c. This derivative value could characterize the change trend of response velocity of electrophoretic particles. Different peak values were observed (Fig. 4c), which indicated the maximum velocity during BTW or WTB process. The corresponding driving voltage was defined as activated voltage, which represented the most efficient voltage for driving EPD comparing to the saturated voltage. As shown in Fig. 4d, the activated voltage decreased monotonically with increment of LC concentration, indicating that electrophoretic particles are more easily driven in the matrix containing LC molecules.

## Discussion

**Figure 5:**
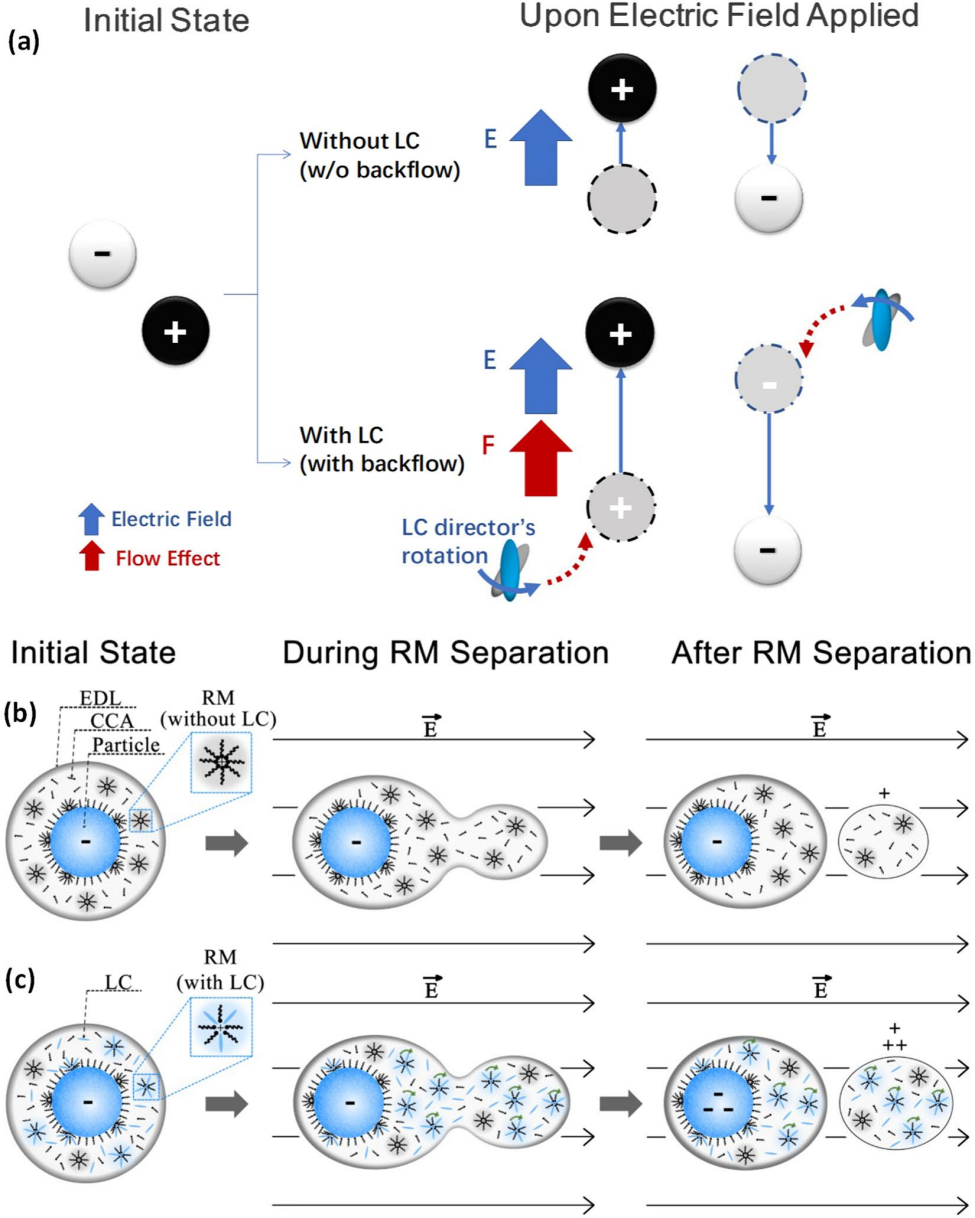
The schematic of (**a**) the backflow effect of liquid crystal molecules and the reverse micelles dielectrophoretic separation process (**b**) without and (**c**) with liquid crystal doping.

The performance improvement of EPD can be attributed to the backflow effect of LC molecules. The movement of electrophoretic particle critically relied on many factors, such as applied voltage, viscosity of suspending liquid and the amount of net charge of particles. In regard to viscosity, LC molecule with anisotropy permittivity could rotate and aligned with the applied electric field which drove its surrounding liquid to create a liquid flow (backflow effect)^16,21^. This rotation and reorientation, acting like Quincke Rotation^11–14^, would induce a hydrodynamic motion in a reduction of the effective viscosity of dispersion^22–24^, that is, the backflow effect of doped LC induced an IER effect (Fig. 5a). It should be noted that the liquid crystal molecules in the Fig. 5a represent the average director of the liquid crystal molecules in the nearby region. The reduced viscosity could decrease the resistance on electrophoretic movement of the particles, which led to the reduction of response time and driving voltage.

**Figure 6:**
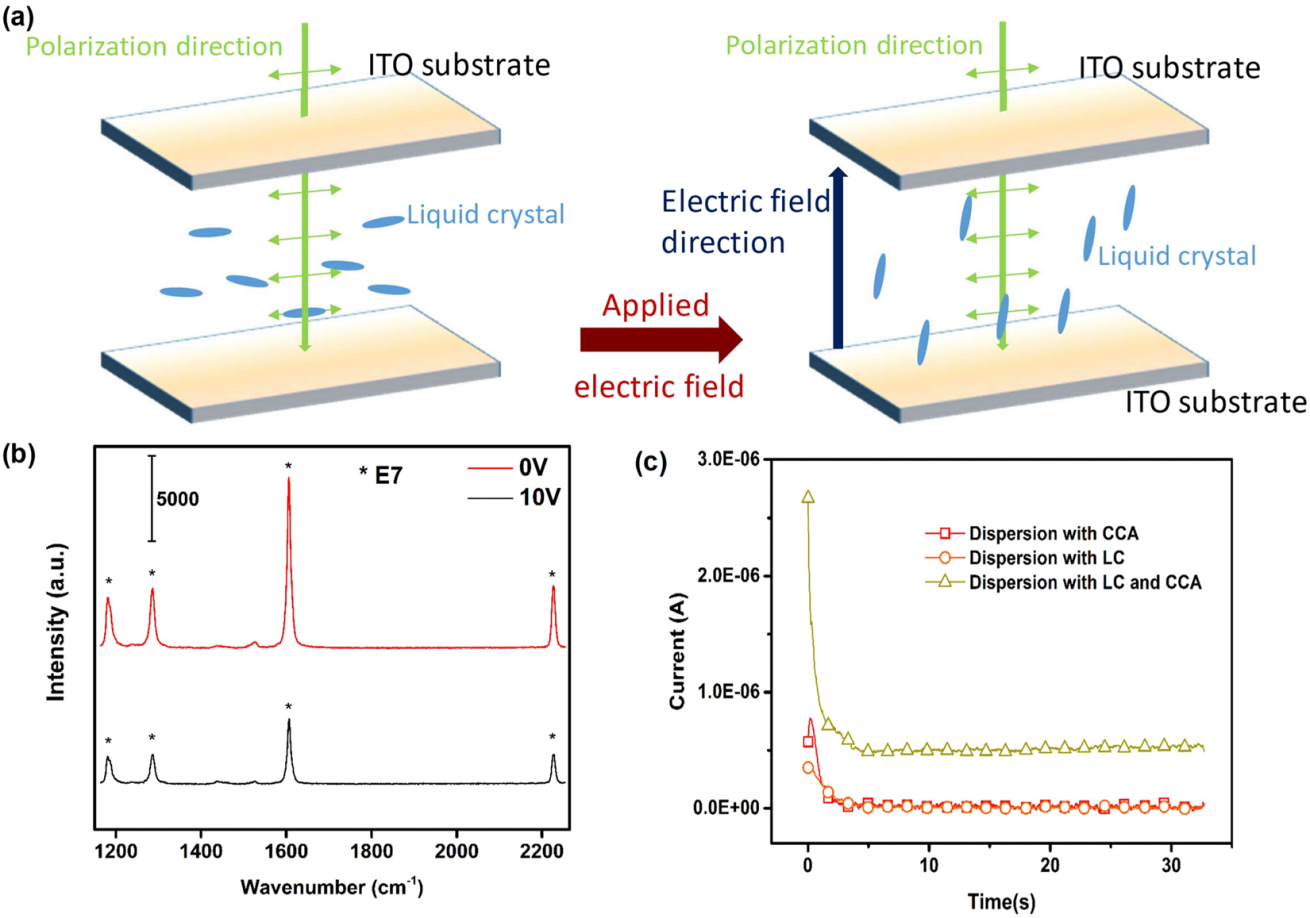
The schematic illustration of (**a**) the changes in Raman intensity for E7 molecules at different orientation and (**b**) the Raman spectra of E7 dissolved in non-polar solvent with and without applied electric field and (**c**) the current of electrophoretic dispersion added with only CCA, only LC and both LC and CCA under 15 V applied voltage.

Raman spectroscopy is a technique for detecting vibration patterns of specific chemical groups, which is able to demonstrate LC’s backflow did occur or not^25,26^ (Fig. 6a). We utilized this method and carried out Raman spectroscopy measurements. A direct current (DC) voltage of 10 V was applied to the test cell mentioned in our manuscript. Figure 6b reveals the Raman spectra of non-polar solvent doping with LC at two applied voltages, 0 V and 10 V, respectively. Then, at 0 V, the orientation of E7 is parallel to the incident laser’s polarized direction and a higher Raman intensity is obtained. When the voltage is 10 V, the intensity of E7 characteristic peaks decreases, indicating that the orientation of E7 molecules changed. The orientation of E7 changes from parallel to perpendicular to the ITO surface when a voltage was applied. Thus, LC can align paralleled to the orientation of applied voltage and enhance the fluid flow through backflow effect^22,23,27^.

Besides the IER effect, the LC molecules could participate in the formation of reverse micelles (RMs) structures and rotate under the applied field that facilitate RMs dielectrophoretic separation from the particles which increase the amount of net charges carried by the particles (Fig. 5b,c). The electrophoretic particles in EPD applications were prepared in non-polar solvent, different from the conventional charging mechanism in polar solvents, EPD needs RMs to transport the charges between species in the solvent media^28^. In the nonpolar solvent, amphipathic CCAs or surfactants would form RMs after reaching the critical micelle concentration (CMC)^29–31^. When the CCAs were added into electrophoretic dispersion, the particles would interact with CCAs or surfactants by several ways, such as preferential adsorption and ion exchanging^32^. After the interaction, particles would carry a few charges on its surface and balanced by an equal amount of charge with an opposite polarity carried by RMs in its surrounding solvent^33^. Due to the competition between the attractive forces caused by Coulombic force between particles and charged RMs and the diffusion force caused by thermal motion^10^, the particles and charged RMs would form an electric double layer (EDL). When an external electric field was applied, dielectrophoresis occurred (Fig. 5b). The particles and RMs, which carried the opposite charges, were driven by external electric field towards the opposite directions. The symmetrical structure of EDL was disturbed and the electric force induced by the external field would be balanced by the attractive forces. Once the external electric field was strong enough to overcome the attractive forces, the RMs started to separate from the particles. The particles would carry net charges and subjected to electrophoretic forces to move towards the electrodes^28^.

The E7 LC molecules consist of a hydrophilic cyano head and a lipophilic alkyl tail which also have amphipathic characteristics, thus, they also participate the formation of RM structures after doped into the dispersion. Because of their better response with external filed as usually used in LCD applications, the LC doped RMs is more likely to be separated from the particles upon external filed caused dielectrophoresis occurs. Moreover, the rotation of the LC molecules would further apply extra force (backflow effect) to the RMs to break the equilibrium between the attractive force of EDL and electric force induced by the external field, and cause the separation of RMs resulting in more net charges carried by particle and more free charged RMs (Fig. 5c). As shown in Fig. 5d, the current of dispersion with both LC and CCA was much larger than the others which indicated that more free charged RMs were separated and contributed to the conductivity. This data indicated that the amount of net charges of particles was increased by LC doping which led to increment of particle velocity and performance improvement. However, if too many RMs were separated from the particles due to the excessive LC doping, the EDL would become too thin to keep the distance between particles and induced the aggregation^34^, which is responsible for the degradation of the optical intensity (Fig. 2b). Therefore, both optical intensity and response time needed to be taken into consideration in order to determine the optimal LC doping concentration of EPD devices.

## Conclusions

In summary, LC molecules were utilized to improve the response time and driving voltage of EPD device. After doped with LC (4 wt%), the optimal WTB and BTW response time of dispersion were 320 ms and 163 ms, respectively, under 15 V applied voltage, and the same intensity of black and white state was achieved when the driving voltage reduced to 7.5 V. In order to probe the variation trend of the response velocity more accurately, an activated voltage was defined as the voltage corresponding to the peak of response curve derivative. The backflow effect of LC molecules was believed to contribute to the performance improvement by inducing the IER effect and facilitating the RMs dielectrophoresis separation, which resulted in the reduction of dispersion viscosity and increment of the net charges carried by particle respectively. This method shows great potential for fast response and low power consumption EPD application.

## Supplementary information

Supplementary information

## Data Availability

The datasets generated during the current study are available from the corresponding authors on reasonable request.
