# Supplementary material for: Backflow Effect Enabling Fast Response and Low Driving Voltage of Electrophoretic E-ink Dispersion by Liquid Crystal Additives
Source: Sci Rep. 2019 Sep 27;9:13981. doi: 10.1038/s41598-019-50382-y (PMC6765022; doi:10.1038/s41598-019-50382-y)
Supplement: Supplementary file 1 — Supplementary information [file 41598_2019_50382_MOESM1_ESM.docx]

**Supplementary INFORMATION**

Backflow Effect Enabling Fast Response and Low Driving Voltage of Electrophoretic E-ink Dispersion by Liquid Crystal Additives

Ya-Di Zhang^1^, Wen-Jie Hu^1^, Zhi-Guang Qiu^1^, Jia-Zhe Xu^1^, Ming-Yang Yang^1^, Yi-Fan Gu^1^, Jin-Xin Cao^1^, Peng Chen^1^, Gui-Shi Liu^2^, Bo-Ru Yang^1*^

^1^State Key Laboratory of Optoelectronic Materials and Technologies, Guangdong Province Key Laboratory of Display Material and Technology, and School of Electronics and Information Technology, Sun Yat-Sen University, Guangzhou, 510006, China

^2^Guangdong Provincial Key Laboratory of Optical Fiber Sensing and Communications, College of Science & Engineering, Jinan University, Guangzhou 510632, China

*paulyang68@me.com

Figure S1. The electro-optical response of pristine electrophoretic dispersion under different driving voltage


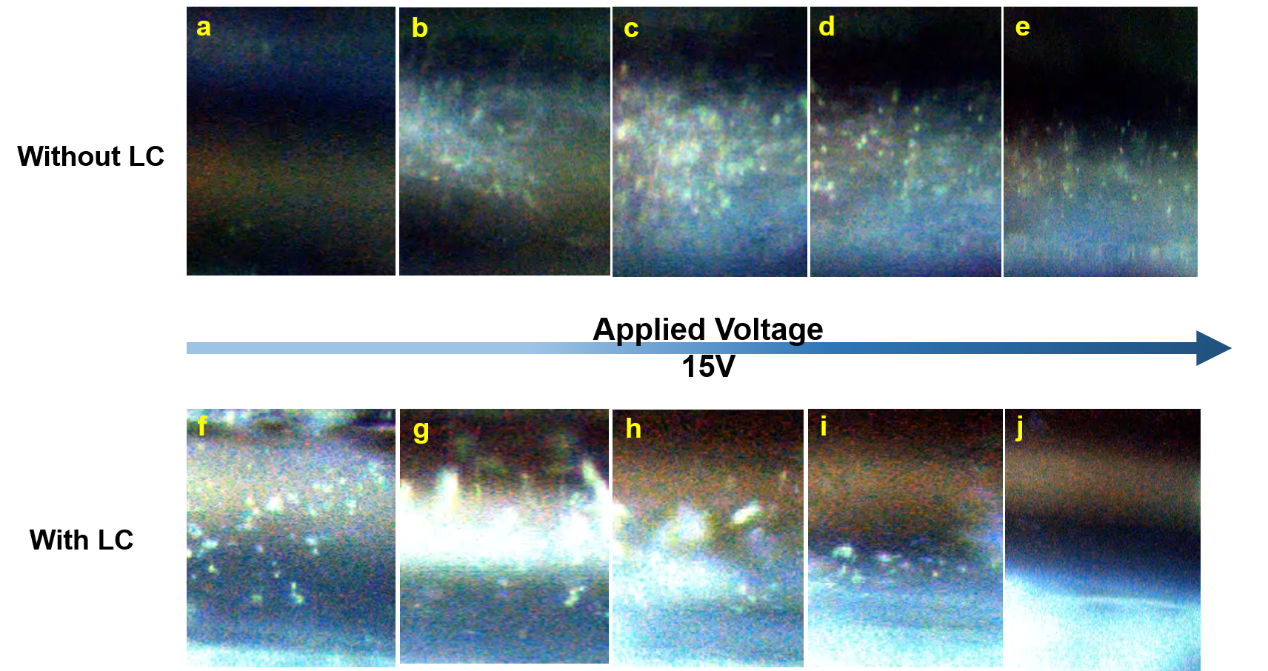


Fig. S2. The gradual changing processes of electrophoretic dispersion upon applying 15V of external field (without LC doped, a-e, and with LC doped, f-j).
